# The psychometric properties of the St George’s Respiratory Questionnaire (SGRQ) in patients with idiopathic pulmonary fibrosis: a literature review

**DOI:** 10.1186/s12955-014-0124-1

**Published:** 2014-08-20

**Authors:** Jeffrey J Swigris, Dirk Esser, Craig S Conoscenti, Kevin K Brown

**Affiliations:** National Jewish Health, Denver, CO USA; Boehringer Ingelheim GmbH, Ingelheim am Rhein, Germany; Boehringer Ingelheim Pharmaceuticals, Inc., Ridgefield, CT USA

**Keywords:** Idiopathic pulmonary fibrosis, Patient-reported outcomes, PROs, St George’s Respiratory Questionnaire, SGRQ, Health-related quality of life, HRQL, Psychometrics, Validity, Reliability

## Abstract

Assessment of health-related quality of life (HRQL) is particularly important in patients with progressive and incurable diseases such as idiopathic pulmonary fibrosis (IPF). The St George’s Respiratory Questionnaire (SGRQ) has frequently been used to measure HRQL in patients with IPF, but it was developed for patients with obstructive lung diseases. The aim of this review was to examine published data on the psychometric performance of the SGRQ in patients with IPF. A comprehensive search was conducted to identify studies reporting data on the internal consistency, construct validity, test-retest reliability, and interpretability of the SGRQ in patients with IPF, published up to August 2013. In total, data from 30 papers were reviewed. Internal consistency was moderate for the SGRQ symptoms score and excellent for the SGRQ activity, impact and total scores. Validity of the SGRQ symptoms, activity, impact and total scores was supported by moderate to strong correlations with other patient-reported outcome measures and with a measure of exercise capacity. Most correlations were moderately strong between SGRQ activity or total scores and forced or static vital capacity, the most commonly used marker of IPF severity. There was evidence that changes in SGRQ domain and total scores could detect within-subject improvement in health status, and differentiate groups of patients whose health status had improved, declined or remained unchanged. Although the SGRQ was not developed specifically for use with patients with IPF, on balance, its psychometric properties are adequate and suggest that it may be a useful measure of HRQL in this patient population. However, several questions remain unaddressed, and further research is needed to confirm the SGRQ’s utility in IPF.

**Electronic supplementary material:**

The online version of this article (doi:10.1186/s12955-014-0124-1) contains supplementary material, which is available to authorized users.

## Introduction

Idiopathic pulmonary fibrosis (IPF) is a specific form of fibrosing interstitial pneumonia characterized by progressive worsening of dyspnea and lung function [1]. In the United States, the annual incidence of IPF has been estimated as 6.8–8.8 cases per 100,000 using narrow case definitions (requiring a definite pattern of Usual Interstitial Pneumonia [UIP] on high-resolution computed tomography [HRCT]), and as 16.3–17.4 cases per 100,000 using broad case definitions (including patients with a possible UIP-pattern on HRCT) [2]. Although IPF has a poor prognosis, with a median survival time from diagnosis of 2 to 3 years, the clinical course of IPF varies considerably [1,3]. Symptoms experienced by patients with IPF include non-productive cough, fatigue and chronic dyspnea, with the latter being the most prominent and disabling [4]. The morbidity associated with IPF has a broad and profound impact on patients’ health-related quality of life (HRQL) [4,5].

As IPF is a progressive disease with no cure, HRQL and other patient-centered outcomes are important endpoints to evaluate in research and clinical practice [6]. Although no disease-specific measure of HRQL has been established as suitable for longitudinal research in patients with IPF, several HRQL instruments (and others, including symptom and generic quality of life questionnaires) have been used [7,8]. Which patient-centered instrument(s) (including HRQL questionnaires) to use in a particular study depends on a number of factors, including the design of the study, the intervention being assessed, the hypotheses being tested, and the characteristics of the comparator group (general population, patients with IPF of different severity, patients with another disease, etc.). In any situation, whether a generic HRQL instrument might perform as well or better than a disease-specific HRQL instrument is uncertain.

In this review, we focused on the St George’s Respiratory Questionnaire (SGRQ). Although originally developed for use in patients with chronic obstructive pulmonary disease (COPD) and asthma [8], it has frequently been used to evaluate HRQL in patients with IPF. The SGRQ is a 50-item questionnaire split into three domains: symptoms (assessing the frequency and severity of respiratory symptoms), activity (assessing the effects of breathlessness on mobility and physical activity), and impact (assessing the psychosocial impact of the disease) [9]. Scores are weighted such that every domain score and the total score range from 0 to 100, with higher scores indicating a poorer HRQL.

The aim of this review was to assess the appropriateness of the SGRQ for measuring HRQL in patients with IPF by examining the evidence relating to the psychometric performance of the SGRQ in this population. A revised version of the SGRQ, the SGRQ-I, has been developed for use in patients with IPF [10]; however, studies assessing this tool are limited, and SGRQ-I data are not covered in this manuscript.

## Methods

### Search strategy and data extraction

**Figure 1:**
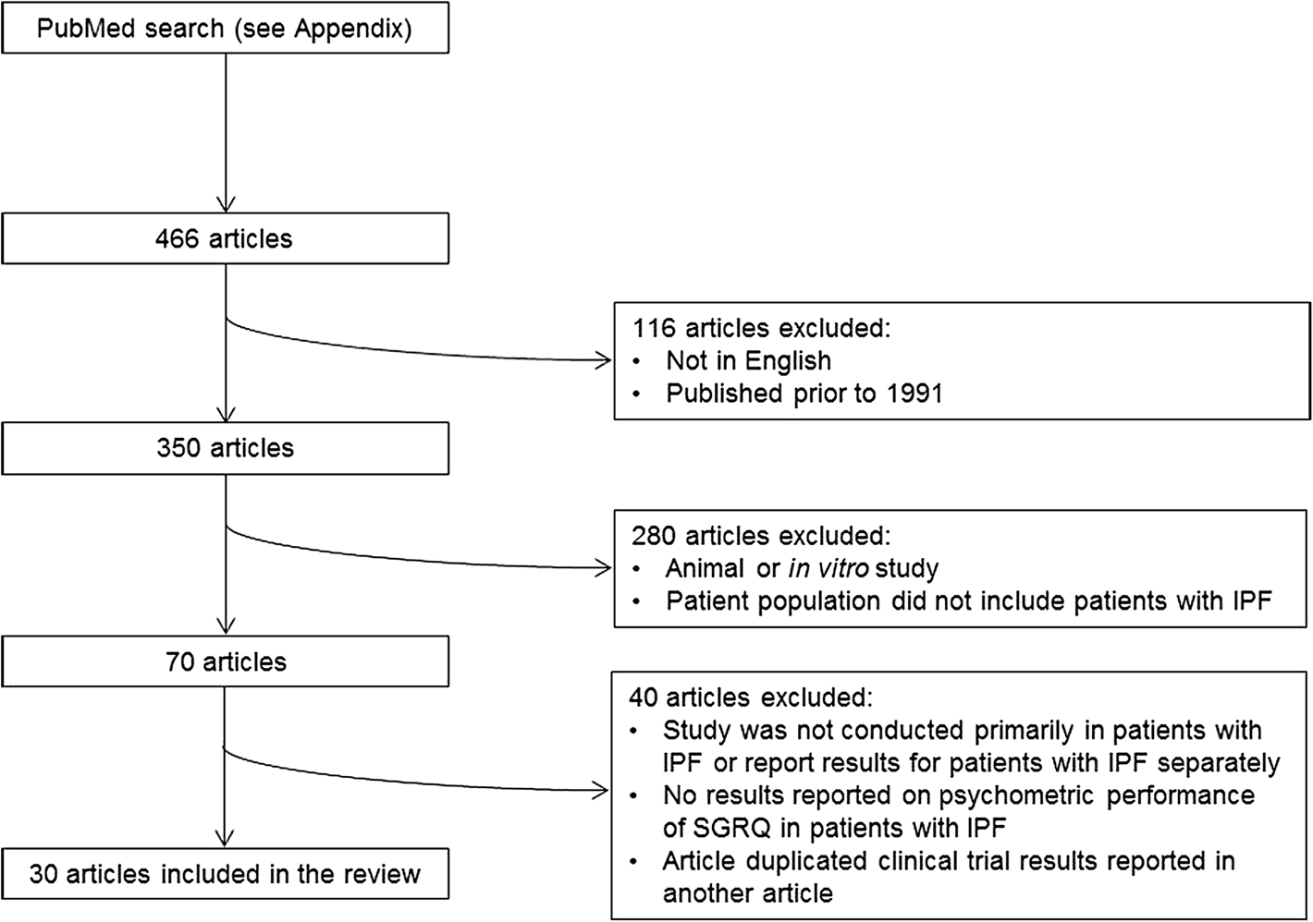
**Selection of articles to be included in the review.**

A comprehensive literature review was conducted to identify articles that evaluated the psychometric properties of the SGRQ in patients with IPF. Following a PubMed search (see Additional file 1), articles were excluded if they were not published between 1 January 1991 (date of first publication of the SGRQ) and 31 August 2013, were not published in English, did not report data on the psychometric properties of the SGRQ in patients with IPF or duplicated clinical trial data reported in another article (Figure 1). Data extracted from the studies included study characteristics (country, duration, design, sample size), participant characteristics (age, gender, time since diagnosis, forced vital capacity [FVC]% predicted, diffusing capacity for carbon monoxide [DL_CO_]% predicted) and results of the psychometric tests.

Articles were selected that assessed any of the following psychometric properties of the SGRQ: internal consistency, convergent validity, known groups validity, test-retest reliability (reproducibility), responsiveness, minimal important difference (MID), and floor and ceiling effects [11]. Internal consistency refers to the degree to which the individual items within an instrument correlate with each other (i.e., tap the same underlying construct). This is determined using Cronbach’s coefficient alpha, with ≥0.70 considered to indicate acceptable internal consistency for a multi-dimensional instrument. Convergent validity describes the degree to which two measures, hypothesized to measure the same construct, correlate. Known groups validity refers to the extent to which scores on an instrument distinguish groups that differ on a key variable, usually clinical in nature. For the described validity measures, correlations were regarded as weak if ≤0.30, moderate if 0.30–0.60, and strong if >0.60 [12]. Test-retest reliability assesses the ability of an instrument to produce consistent scores over repeated measurements in patients who are clinically stable. Responsiveness assesses the ability of an instrument to detect change in individuals who are hypothesized to have changed on the underlying construct (HRQL) and who are known to have experienced change in clinical status. MID estimates identify the smallest difference in the score on an instrument that patients perceive as important. Floor and ceiling effects are limitations that occur when an individual scores at the extremes of an instrument; if a patient’s score is the lowest or highest possible value, the instrument is unable to detect a reduction or increase, respectively.

## Results

A total of 30 papers were included in the review (Figure 1; Table 1).

**Table 1:** **Studies included in this review**

**Study**	**Study type**	**Experimental treatment**	**Country**	**Sample size** ^**1**^		**Disease duration (mo) mean (SD)**	**Baseline SGRQ score** ^**2**^	**Baseline spirometric values** ^**2**^	
				**All**	**IPF**			**FVC% predicted**	**DL**_**CO**_ **% predicted**
Antoniou *et al.*, 2006 [13]	RCT	Interferon gamma b	Greece	50	50	T = 49.4 (24.3) C = 42.7 (16.8)	--	T = 71.8 (15.0) C = 70.7 (17.7)	--
Berry *et al.*, 2012 [14]	Secondary validation study	n/a	US	405	239	--	--	66.0 (52–78)	--
Chang *et al.*, 1999 [15]	Standalone validation study	n/a	US	50	33	--	Total = 38.9 [28.7–55.2] Symptoms domain = 50.5 [31.5–69.8], Activity domain = 54.4 [39.9–72.9], Impact domain = 28.4 [18.8–45.1]	65.0 (49.0–81.0)	49.0 (36.5–59.8)
du Bois *et al.*, 2011 [16]	RCT	Interferon gamma b	Multi-national	822	822	--	Total = 41.8 (18)	72.5 (12.7)	47.4 (9.2)
Han *et al.*, 2013 [17]	RCT	Sildenafil	US	119	119	20.4	--	56.9	26.0
Horton *et al.*, 2012 [18]	RCT	Thalidomide	US	23	23	20.5 (3–59)	Total = 57.4 (18.8) Symptoms domain = 67.7 (19.7), Activity domain = 64.3 (22.7), Impact domain = 48.1 (20.7)	70.4 (13.7)	57.4 (14.4)
King, Jr. *et al.*, 2008 [19]	RCT	Bosentan	Multi-national	158	158	T = 12.2 (12.2) C = 12.1 (12.0)	T-Total = 45.7 (18.1), C-Total = 45.2 (19)	T = 65.9 (10.5), C = 69.5 (12.6)	T = 42.3 (9.5), C = 41.4 (9.5)
King, Jr. *et al.*, 2009 [20]	RCT	Interferon gamma b	Multi-national	826	826	–	T-Total = 41.6 (17.9), C-Total = 42.4 (18.2)	T = 72.2 (12.3), C = 73.1 (13.4)	T = 47.4 (9.2), C = 47.3 (9.3)
Lechtzin *et al.*, 2013 [21]	RCT	Thalidomide	US	24	24	--	Total = 57.4 (18.8), Symptoms domain = 67.7 (19.7), Activity domain = 64.3 (22.7), Impact domain = 48.1 (20.7)	70.4 (13.7)	57.4 (14.4)
Mishra *et al.*, 2011 [22]	Within-subject trial	Oral doxycycline	India	6	6	--	Total = 50.90 (8.38)	n/a	n/a
Naji *et al.*, 2006 [23]	Within-subject trial	Pulmonary rehabilitation	Ireland	26	19	--	Total = 48 (27.6, 67.9)	66.7 (20.7)	42.5 (14)
Nishiyama *et al.*, 2005 [24]	Standalone validation study	n/a	Japan	41	41	--	Total = 35.7 (20.6) [range 1.6–77.6], Symptoms domain = 40.1 (24.6) [range 4.4–85.6], Activity domain = 44.5 (26.7) [range 0–93.9], Impact domain = 28.9 (19.8) [range 0–77.0]	n/a	n/a
Nishiyama *et al.*, 2008 [25]	RCT	Pulmonary rehabilitation	Japan	28	28	--	T-Total = 50.2 (16.3), T-Symptoms domain = 56.4 (22.3), T-Activity domain = 64.7 (17.1), T-Impact domain = 39.7 (17.6), C-Total = 37.8 (22.7), C-Symptoms domain = 38.0 (25.8), C-Activity domain = 50.4 (26.2), C-Impact domain = 29.9 (23.7)	T = 66.1 (13.2), C = 68.7 (19.5)	T = 59.4 (16.7), C = 48.6 (16.7)
Noth *et al.*, 2012 [26]	RCT	Warfarin	US	145	145	T = 21.6, C = 25.2	T-Total = 46.2 (18.0), C-Total = 50.1 (17.2)	T = 58.9 (16.2), C = 58.7 (16.1)	T = 33.8 (12.4), C = 34.6 (13.4)
Patel *et al.*, 2012 [27]	Standalone validation study	n/a	UK	173	49	48.0	--	82 (34–143)	--
Peng *et al.*, 2008 [28]	Standalone validation study	n/a	China	68	68	14.0 (14.0)	Total = 54 (15), Symptoms domain = 65 (16), Activity domain = 56 (15), Impact domain = 49 (19)	66 (18)	54 (16)
Raghu *et al.*, 2004 [29]	RCT	Interferon gamma b	Multi-national	330	330	--	--	T = 63.9 (10.7), C = 64.1 (11.3)	--
Raghu *et al.*, 2008 [30]	RCT	Etanercept	Multi-national	88	88	T = 14.7 (19.8), C = 12.3 (13.6)	T-Total = 40.8 (18.1), C-Total = 42.9 (19.4)	T = 64.7 (14.1), C = 63.0 (12.7)	T = 36.3 (12.6), C = 36.9 (10.8)
Raghu *et al.*, 2013 [31]	RCT	Ambrisentan	Multi-national	492	492	T = 13.2, C = 10.8	T-Total = 44.5 (21.6), C-Total = 40.5 (21.1)	T = 68.7 (13.1), C = 69.9 (13.8)	T = 42.0 (13.8), C = 45.6 (13.3)
Rammaert *et al.*, 2009 [32]	Within-subject trial	Pulmonary rehabilitation	France	13	13	--	--	67 (14)	32 (13)
Richeldi *et al.*, 2011 [33]	RCT	Nintedanib (BIBF 1120)	Multi-national	428	428	T, 50 mg qd = 16.8 (15.6), T, 50 mg bid = 13.2 (14.4), T, 100 mg bid = 14.4 (14.4), T, 150 mg bid = 12 (14.4), C = 16.8 (18)	T, 50 mg qd-Total = 43.7 (17.5), T, 50 mg bid-Total = 42.5 (17.0), T, 100 mg bid-Total = 43.7 (16.6), T, 150 mg bid-Total = 40.1 (18.3), C-Total = 41.2 (17.9)	T, 50 mg qd = 80.4 (17.8), T, 50 mg bid = 79.8 (15.8), T, 100 mg bid = 85.5 (19.2), T, 150 mg bid = 79.1 (18.5), C = 81.7 (17.6)	–
Swigris *et al.*, 2010 [34]	Secondary validation study	Bosentan	Multi-national	158	158	--	Total = 44.8 (19.5), Symptoms domain = 50.1 (21.9), Activity domain = 60.6 (22.8), Impact domain = 33.7 (20.6)	67.0 (12.8)	40.98 (10.1)
Swigris *et al.*, 2012 [35]	Secondary validation study	Sildenafil	US	180	180	24.0	Activity domain = 69.6 (17.6)	56.8 (14.2)	26.3 (6.1)
Tzanakis *et al.*, 2005 [36]	Standalone validation study	n/a	Greece	25	25	31.2	Total = 37.7 (18.9), Symptoms domain = 55.9 (25.3), Activity domain = 36.2 (21.4), Impact domain = 29.6 (21)	68.8 (16)	--
Tzouvelekis *et al.*, 2013 [37]	Within-subject trial	Adipose-derived stromal cells	Greece	14	14	--	--	--	--
Verma *et al.*, 2011 [38]	Standalone validation study	n/a	Canada	137	137	–	Total = 63.4 (3.7–96.3), Symptoms domain = 59.8 (0–97.2), Activity domain = 81.6 (6.0–99.5), Impact domain = 54.1 (0–96.4)	61.7 (19.8)	49.5 (17.9)
Yorke *et al.*, 2010^3^ [10]	Secondary validation study	Bosentan	Multi-national	158	158	--	--	61.0 (12.2)	--
Yorke *et al.*, 2011 [39]	Standalone validation study	n/a	Multi-national	101	67	--	Total = 53 (24), Symptoms domain = 61 (23), Activity domain = 65 (30), Impact domain = 41 (24)	77 (19.5)	51.6 (21)
Zimmermann *et al.*, 2007 [40]	Standalone validation study	n/a	Brazil	22	22	--	Total = 48.4 (17.9), Symptoms domain = 46.4 (20.3), Activity domain = 62.4 (19), Impact domain = 43.6 (20.9)	70.4 (19.4)	41.5 (16.2)
Zisman *et al.*, 2010 [41]	RCT	Sildenafil	US	180	180	T = 24.4, C = 22.4	T-Total = 54.55 (16.46), C-Total = 51.72 (15.86)	T = 54.9 (14.00), C = 58.7 (14.12)	--

^1^Sample size reported represents the population in which efficacy was assessed. RCT = randomized controlled trial; T = treatment group; C = comparator group; qd = once daily; bid = twice daily. ^2^Mean (SD) or median [interquartile range] are reported based on availability. ^3^Data reported refer to the original version of the SGRQ, not the SGRQ-I.

### Internal consistency

Data from a clinical trial of bosentan have been used to determine the internal consistency of the SGRQ in patients with IPF. Cronbach’s alpha was 0.66 for the symptoms score and ≥0.84 for each of the SGRQ activity, impact and total scores [10,34].

### Convergent validity

Convergent validity was evaluated by extracting cross-sectional and longitudinal correlations between SGRQ scores and other patient-reported outcome measures (Table 2), an assessment of exercise capacity (Table 3), pulmonary function tests (PFTs) or partial pressure of arterial oxygen (Table 4), and assessments of fibrotic abnormalities on HRCT (Table 5).

**Table 2:** **Correlation coefficients between SGRQ scores and other patient-reported assessments of health status**

	**Measure**	**Scale**	**Correlation with SGRQ symptoms domain score**	**Correlation with SGRQ activity domain score**	**Correlation with SGRQ impact domain score**	**Correlation with SGRQ total score**
**Cross-sectional studies**						
Chang *et al.*, 1999 [15]	Borg Dyspnea Index					0.56^†^
Lechtzin *et al.*, 2013 [21]	CQLQ	Total	0.72*	0.72^‡^	0.81^‡^	0.79^‡^
		Physical complaints	0.50*	0.72^‡^	0.71^‡^	0.77^‡^
		Psychological issues	0.29	0.40	0.62^†^	0.54*
		Functional ability	0.53*	0.54*	0.66^†^	0.66^†^
		Emotional well-being	0.19	0.42	0.57^†^	0.50*
		Extreme physical complaints	0.38	0.34	0.63^†^	0.54*
		Personal safety fears	0.05	0.23	0.45*	0.34
Nishiyama *et al.*, 2005 [24]	BDI		−0.55^‡^	−0.77^§^	−0.53^‡^	−0.69^§^
Patel *et al.*, 2012 [27]	K-BILD	Total	−0.67^†^	−0.79^†^	−0.87^†^	−0.89^†^
		Psychological	−0.60^†^	−0.67^†^	−0.80^†^	−0.79^†^
		Breathlessness	−0.59^†^	−0.84^†^	−0.80^†^	−0.86^†^
		Chest	−0.65^†^	−0.64^†^	−0.79^†^	−0.78^†^
Peng *et al.*, 2008 [28]	Dyspnea score		NS	0.58^§^	0.30^†^	0.38^‡^
Swigris *et al.*, 2012 [35]	UCSD-SOBQ			0.80^§^		
Yorke *et al.*, 2010^1^ [10]	BDI		−0.39^§^	−0.72^§^	−0.61^§^	−0.68^§^
	SF-36 PCS		−0.52^§^	−0.74^§^	−0.63^§^	−0.71^§^
	Borg Dyspnea Index		0.35^§^	0.45^§^	0.40^§^	0.45^§^
Yorke *et al.*, 2011 [39]	D-12		0.57^‡^	0.78^‡^	0.75^‡^	0.79^‡^
Zimmermann *et al.*, 2007 [40]	BDI		−0.62*	−0.75*	−0.63*	−0.72*
**Longitudinal studies**						
Nishiyama *et al.*, 2008 [25]	Δ BDI					−0.29
Peng *et al.*, 2008 [28]	Δ Dyspnea score		NS	0.59^†^	0.56^†^	0.45^†^

BDI = Baseline Dyspnea Index; CQLQ = Cough Quality of Life Questionnaire; D-12 = Dyspnea-12; K-BILD = King’s Brief Interstitial Lung Disease questionnaire; SF-36 PCS = SF-36 Physical Component Summary; UCSD-SOBQ = University of California San Diego Shortness of Breath Questionnaire; Δ = change.

*p < 0.05; ^†^p < 0.01; ^‡^p < 0.001; ^§^p < 0.0001; NS = non-significant. ^1^Data reported refer to the original version of the SGRQ, not the SGRQ-I.

**Table 3:** **Correlation coefficients between SGRQ scores and the 6MWD as a measure of exercise capacity**

	**Measure**	**Correlation with SGRQ symptoms domain score**	**Correlation with SGRQ activity domain score**	**Correlation with SGRQ impact domain score**	**Correlation with SGRQ total score**
**Cross-sectional studies**					
Chang *et al.*, 1999 [15]	6MWD				−0.66^†^
du Bois *et al.*, 2011 [16]	6MWD				−0.26^‡^
Peng *et al.*, 2008 [28]	6MWD	−0.32^†^	−0.43^‡^	−0.41^‡^	−0.45^‡^
Yorke *et al.*, 2010^1^ [10]	6MWD	−0.14	−0.32^§^	−0.24^†^	−0.28^†^
Yorke *et al.*, 2011 [39]	6MWD	−0.32^†^	−0.54^†^	−0.47^†^	
Zimmermann *et al.*, 2007 [40]	6MWD	−0.41	−0.72*	−0.63*	−0.72*
**Longitudinal studies**					
du Bois *et al.*, 2011 [16]	Δ 6MWD				−0.231^‡^
Nishiyama *et al.*, 2008 [25]	Δ 6MWD				−0.43*
Peng *et al.*, 2008 [28]	Δ 6MWD	NS	−0.43	−0.46	−0.41^†^

6MWD = Distance covered in 6-minute walk test; Δ = change.

*p < 0.05; ^†^p < 0.01; ^‡^p < 0.001; ^§^p < 0.0001; NS = non-significant. ^1^Data reported refer to the original version of the SGRQ, not the SGRQ-I.

**Table 4:** **Correlation coefficients between SGRQ scores, pulmonary function tests and arterial blood gas analysis**

	**Lung function measure**	**Correlation with SGRQ symptoms domain score**	**Correlation with SGRQ activity domain score**	**Correlation with SGRQ impact domain score**	**Correlation with SGRQ total score**
Chang *et al.*, 1999 [15]	DL_CO_% predicted				−0.55^†^
	FEV_1_% predicted				−0.46^†^
	FVC% predicted				−0.45^†^
	TLC% predicted				−0.36^†^
Nishiyama *et al.*, 2005 [24]	PaO_2_	−0.21	−0.48^†^	−0.29	−0.37*
	SpO_2_	−0.38*	−0.48^†^	−0.22	−0.37*
	TLC	−0.48^†^	−0.38*	−0.21	−0.36*
	TL_CO_	−0.32*	−0.45^†^	−0.27	−0.39*
	VC	−0.35*	−0.36*	−0.15	−0.30
Peng *et al.*, 2008 [28]	DL_CO_% predicted	−0.46^§^	−0.46^§^	−0.34^†^	−0.44^‡^
	FEV_1_% predicted	NS	−0.53^§^	−0.34^†^	−0.42^§^
	PaO_2_	NS	−0.54^‡^	NS	−0.32^†^
	TLC% predicted	−0.50^§^	−0.61^§^	−0.52^§^	−0.62^§^
	VC% predicted	NS	−0.59^§^	−0.35^†^	−0.47^§^
Tzanakis *et al.*, 2005 [36]	FEV_1_% predicted				−0.50^†^
	PaO_2_ (at rest)				−0.51^†^
	PaO_2_ (at exertion)				−0.60^†^
	TLC% predicted				−0.55^†^
Yorke *et al.*, 2010^1^ [10]	FVC% predicted	−0.27^†^	−0.31^§^	−0.30^§^	−0.34^§^
	TL_CO_% predicted	−0.23^†^	−0.34^§^	−0.38^§^	−0.38^§^
Yorke *et al.*, 2011 [39]	DL_CO_%	−0.16	−0.37^†^	−0.28*	
	FVC%	−0.13	−0.16	−0.24*	
Zimmermann *et al.*, 2007 [40]	DL_CO_% predicted	−0.41	−0.32	−0.39	−0.47*
	FEV_1_% predicted	−0.08	−0.57*	−0.52*	−0.57*
	TLC% predicted	−0.37	−0.65*	−0.58*	−0.66*
	VC% predicted	−0.14	−0.54*	−0.61*	−0.56*

DL_CO_ = diffusion capacity of the lung for carbon monoxide; FEV_1_ = forced expiratory volume in 1 second; FVC = forced vital capacity; PaO_2_ = partial pressure of oxygen dissolved in arterial blood; TLC = total lung capacity, TL_CO_ = transfer factor of the lung for carbon monoxide; VC = vital capacity.

*p < 0.05; ^†^p < 0.01; ^‡^p < 0.001; ^§^p < 0.0001; NS = non-significant. ^1^Data reported refer to the original version of the SGRQ, not the SGRQ-I.

**Table 5:** **Correlation coefficients between SGRQ scores and extent of fibrosis on HRCT**

**Study**	**HRCT measure**	**Correlation with SGRQ symptoms domain score**	**Correlation with SGRQ activity domain score**	**Correlation with SGRQ impact domain score**	**Correlation with SGRQ total score**
Peng *et al.*, 2008 [28]	CT-alv	0.41^†^	NS	0.34*	0.39^†^
	CT-fib	NS	0.37*	NS	NS
	CT-tot	0.36*	0.39^†^	0.35*	0.42^†^

CT-alv = ground glass opacity; CT-fib = interstitial opacity; CT-tot = total.

*p < 0.01; ^†^p < 0.001; NS = non-significant.

### Patient-reported outcome measures

In nine studies, investigators provided information on the correlation between SGRQ scores and other patient-reported outcome measures (BDI [Baseline Dyspnea Index], D-12 [Dyspnea-12], K-BILD [King’s Brief Interstitial Lung Disease questionnaire], UCSD-SOBQ [University of California San Diego Shortness of Breath Questionnaire], CQLQ [Cough Quality of Life Questionnaire], a single-item dyspnea assessment, SF-36 Physical Component Summary score [SF-36 PCS] and the Borg Dyspnea Index) (Table 2). Moderate to strong correlations were observed between the SGRQ total score and the total scores on these instruments (Table 2). In general, moderate to strong correlations were observed between SGRQ domain scores and the total scores on these instruments. Likewise, moderate to strong correlations were observed between SGRQ domain or total scores and the total, physical complaints, extreme physical complaints, and functional ability sub-scale scores of the CQLQ (r = 0.34 to 0.81) [21], the total and sub-scale scores of the K-BILD (r = -0.59 to -0.89) [27], the SF-36 PCS, a composite score measuring overall physical health (r = -0.52 to -0.74) [10] and the Borg Dyspnea index (r = 0.35 to 0.56) [10,15]. For most measures and their sub-scales, correlations were weakest with the SGRQ symptoms score (when compared with other SGRQ domains or the total score).

In two studies, investigators evaluated correlations between SGRQ change scores and change scores from other patient-reported outcome measures (Table 2). In one study, correlations were moderately strong between change scores for the SGRQ activity, impact and total scores and change scores from the single-item dyspnea assessment (r = 0.59, 0.56 and 0.45, respectively) [28]. In the other study, investigators found that the correlation between the BDI change score and SGRQ total change score was -0.29 and not significant [25]. However, the BDI was designed to measure dyspnea severity at a single point in time and not to measure change in dyspnea severity [42].

### Measures of exercise capacity

Correlation coefficients between SGRQ scores and a measure of exercise capacity are presented in Table 3. Distance covered during the 6-minute walk test (6MWD) is frequently used as a measure of exercise capacity in patients with IPF, and change in 6MWD has been shown to be a predictor of mortality in these patients [16]. In five cross-sectional studies in patients with IPF, investigators examined the relationship between the SGRQ total score and 6MWD. The strength of these correlations was moderate to strong in three (-0.45 to -0.72) [15,28,40] and weak in two (-0.26 and -0.28) [10,16] studies. In four cross-sectional studies, investigators examined the relationship between the SGRQ domain scores and the 6MWD [10,28,39,40]; the strength of these correlations was moderate to strong for the activity score in all four studies (r = -0.32 to -0.72), moderate to strong for the impact score (r = -0.41 to -0.63) and moderate for the symptoms score (r = -0.32 to -0.41) in three studies. In three studies, investigators examined the relationship between change scores for the SGRQ total and change in 6MWD;[16,25,28]correlation coefficients ranged from -0.23 to -0.43.

### Pulmonary function tests and arterial blood gas analysis

Table 4 presents correlations between SGRQ scores and either PFTs or arterial blood gas analysis in patients with IPF. All correlations between the SGRQ total score and these variables were moderate to strong (r = -0.30 to -0.66, and p < 0.05 for all but one). There were moderate to strong correlations between the SGRQ activity score and the majority of pertinent PFT results (e.g., FVC or DL_CO_) or arterial blood gas analysis in all studies, while correlations between the SGRQ symptoms or impact domain scores and these variables were generally weak to moderate. Results for FVC, the lung function parameter regarded as the most statistically useful physiological indicator of IPF severity, and the one most frequently used as a primary endpoint in contemporary clinical trials, were weakly to moderately correlated with SGRQ total and domain scores (r = -0.34 to -0.45 for the SGRQ total and -0.13 to -0.31 for the SGRQ domains).

### HRCT

In one study of patients with IPF, investigators assessed correlations between SGRQ scores and the extent of fibrotic abnormalities on HRCT (degree of ground-glass opacity [CT-alv], interstitial opacity [CT-fib], and both [total score]) (Table 5). Correlations were moderately strong between the SGRQ symptoms, impact and total scores and CT-alv or total scores (r = 0.34 to 0.42) and moderately strong between the SGRQ activity score and both the CT-fib and total scores (r = 0.37 to 0.39) [28].

### Known groups validity

Although there are no well-established categories of disease severity in IPF, it may be hypothesized that patients receiving supplemental oxygen represent patients with more severe disease. In two studies, investigators found that SGRQ total scores were worse in patients using supplemental oxygen versus those not using supplemental oxygen [15,38]. In one study by Chang and colleagues, the magnitude of difference between patients using versus not using oxygen was 4.7 (p < 0.05) [15].

### Test-retest reliability (reproducibility)

No studies were found that reported data on the test-retest reliability of the SGRQ in patients with stable IPF.

### Minimal important difference

A triangulation approach has been used to determine an MID estimate for SGRQ scores in patients with IPF [34]. Using both distribution- and anchor-based approaches (using FVC, DL_CO_ and the TDI as anchors), the MID for the SGRQ symptoms, activity, impact and total scores was 8, 5, 7 and 7 respectively.

### Responsiveness

The responsiveness of the SGRQ domain and total scores has been assessed in one study [34]. Using data from a randomized placebo-controlled trial of bosentan, investigators assessed the ability of the SGRQ to discriminate among IPF patients who had experienced an improvement, decline, or no change in disease status over 6 months, as defined by three clinical anchors (change in FVC, DL_CO_, transition dyspnea index [TDI]). With the exception of the SGRQ symptoms score when DL_CO_ was the anchor, changes in SGRQ domain and total scores differed significantly between patients who had declined, remained stable, or improved. [34]. Change scores from the SGRQ total and its domains were reported for the DL_CO_ and TDI response categories and ranged from +3 to +13, +1 to -5, and 0 to -12 for patients that declined, remained stable, or improved, respectively. The impact domain discriminated best between all categories of change for all three anchors [34].

### SGRQ as an endpoint

In sixteen trials, investigators used the SGRQ domain and/or total scores as outcome variables. In four trials, investigators evaluated the within-subject change in SGRQ total score from baseline to end of treatment [22,23,32,37] (Table 6). In all four, improvements were observed in exercise endurance or FVC; among these, in three there was a significant decrease in SGRQ total score from baseline to end of treatment (8–24 weeks).

**Table 6:** **Changes in SGRQ scores in within-subject clinical trials**

**Study**	**Treatment under investigation**	**Treatment duration**	**Sample size**		**SGRQ total score** ^**1**^		**Effect size**	**p-value** ^**2**^
			**Total**	**IPF**	**Baseline**	**Post-treatment**		
Mishra *et al.*, 2011 [22]	Oral doxycycline	24 weeks	6	6	50.90 (8.38)	18.40 (6.39)	3.88	<0.001
Naji *et al.*, 2006 [23]	Pulmonary rehabilitation	8 weeks	26	19	48.3 [21.5, 82]	39.5 [17.4, 69.4]	0.41	<0.10
Rammaert *et al.*, 2009 [32]	Pulmonary rehabilitation	8 weeks	13	13	–	–	–	NS
Tzouvelekis *et al.*, 2013 [37]	Endobronchial infusion of adipose-derived stromal cells	6 months	14	14	35.1 (6.8)	27.8 (5.6)	1.07	<0.05

^1^Mean (SD) or median [range] are reported based on availability.

^2^p-value for test of statistical significance between SGRQ score at baseline and post-treatment.

In the remaining 12 trials, investigators assessed whether the SGRQ domain and/or total scores differed between active and placebo groups (Table 7). In four of these [13,17,18,25], statistically significant between-group differences for the primary endpoint coincided with statistically significant between-group differences in at least one SGRQ total or domain score (range of between-groups difference in SGRQ total score: -6.1 to -13.4). Six studies [17,20,26,29-31] reported a lack of statistically significant treatment effect in the primary endpoint or SGRQ scores (range of between-groups difference in SGRQ total score reported in three studies: -0.5 to -3.0; scores were not reported in three studies). In three studies [19,33,41], the primary endpoint was not met, but the SGRQ total or domain scores were significantly different between treatment groups (range of between-groups difference in SGRQ total score: -3.3 to -6.1).

**Table 7:** **Changes in SGRQ scores in randomized controlled trials**

**Study**	**Treatment duration**	**Sample size, IPF**	**Randomized groups**	**Change from baseline in SGRQ symptoms domain score**	**Change from baseline in SGRQ activity domain score**	**Change from baseline in SGRQ impact domain score**	**Change from baseline in SGRQ total score**
Antoniou *et al.*, 2006 [13]	12 months	50	Interferon gamma b	−13.2 [21.4,5.0]	−4.8 [-12.7, 3.0]	−1.9 [-9.2, 5.4]	−4.7 [-11.4, 2.0]
			Colchicine	7.5 [-4.5, 19.5]	4.7 [-12.1, 22.0]	4.1 [-6.4, 14.6]	4.8 [-5.9, 15.5]
			p-value^2^	0.01	NS	NS	NS
Han *et al.*, 2013 [17]	12 weeks	22	Sildenafil (with RVSD)	–	–	–	–
			Placebo (with RVSD)	–	–	–	–
			Difference^1^	−28.0 [-41.7, -14.4]	−5.6 [-16.1, 5.0]	−14.0 [-25.6, -2.4]	−13.4 [-22.7, -4.2]
			p-value^2^	<0.0001	NS	0.02	0.005
		97	Sildenafil (without RVSD)	–	–	–	–
			Placebo, (without RVSD)	–	–	–	–
			Difference^1^	−3.8 [-10.7, 3.0]	−4.1 [-9.2, 1.1]	−1.8 [-7.5, 3.9]	−3.0 [-7.6, 1.7]
			p-value^2^	NS	NS	NS	NS
Horton *et al.*, 2012 [18]	12 weeks	23	Thalidomide	–	–	–	–
			Placebo	–	–	–	–
			Difference^1^	−12.1 [22.2,2.0]	−3.3 [-9.8, 3.2]	−13.1 [-19.7, -6.6]	−11.7 [-18.6, -4.8]
			p-value^2^	0.018	NS	<0.001	0.001
King, Jr. *et al.*, 2008 [19]	6 months^4^	158	Bosentan	–	–	–	–
			Placebo	–	–	–	–
			Difference^1^	–	–	–	−3.3 (2.6)
			p-value^2^	–	–	–	0.034
King, Jr. *et al.*, 2009 [20]	77 weeks	826	Interferon gamma b	--	--	--	5.7 (13.5)
			Placebo	–	–	–	6.2 (14.3)
			p-value^2^	–	–	–	NS
Nishiyama *et al.*, 2008 [25]	10 weeks	28	Pulmonary rehabilitation	–	–	–	–
			No pulmonary rehabilitation	–	–	–	–
			Difference^1^	−5.7 [-18.7, 7.2]	−5.8 [-14.7, 3.1]	−6.2 [-12.8, 0.3]	−6.1 [-11.7, 0.5]
			p-value^2^	NS	NS	NS	<.05
Noth *et al.*, 2012 [26]	28 weeks	145	Warfarin	–	–	–	–
			Placebo	–	–	–	–
			p-value^2^	–	–	–	NS
Raghu *et al.*, 2004 [29]	48 weeks	330	Interferon gamma 1b	–	–	–	–
			Placebo	–	–	–	–
			p-value^2^	–	–	–	NS
Raghu *et al.*, 2008 [30]	48 weeks	88	Etanercept	–	–	–	--
			Placebo	–	–	–	--
			Difference^1^	–	–	–	--
			p-value^2^	NS	NS	NS	NS
Raghu *et al.*, 2013 [31]	48 weeks	492	Ambrisentan	–	–	–	4.7
			Placebo	–	–	–	3.0
			p-value^2^	–	–	–	NS
Richeldi *et al.*, 2011 [33]	12 months	431	Nintedanib 50 mg qd	3.39 (2.51)	7.39 (1.96)	3.71 (2.04)	4.67 (1.78)
			Nintedanib 50 mg bid	2.11 (2.34)	3.54 (1.82)	1.73 (1.90)	2.18 (1.65)
			Nintedanib 100 mg bid	2.33 (2.35)	3.00 (1.83)	0.79 (1.91)	1.48 (1.66)
			Nintedanib 150 mg bid	−3.14 (2.40)	0.32 (1.89)	−0.14 (1.97)	−0.66 (1.71)
			Placebo	6.45 (2.45)	7.48 (1.91)	4.21 (1.99)	5.46 (1.73)
			p-value^3^	<0.005	<0.005	–	<0.01
Zisman *et al.*, 2010 [41]	12 weeks	180	Sildenafil	−3.58 [-7.02, -0.13]	−1.15 [-3.68, 1.38]	−0.88 [-3.78, 2.02]	−1.64 [-3.91, 0.64]
			Placebo	2.15 [-1.30, 5.61]	2.49 [0.00, 4.99]	2.82 [-0.03, 5.67]	2.45 [0.17, 4.72]
			Difference^1^	−5.73 [-10.61, -0.85]	−3.64 [-7.20, -0.09]	−3.70 [-7.76, 0.37]	−4.08 [-7.30, -0.86]
			p-value^2^	0.02	.04	NS	.01

RVSD = right ventricular systolic dysfunction.

^1^Difference in change from baseline between treatment groups, mean [95% CI].

^2^Test of statistical significance for the difference in mean change from baseline between groups.

^3^Test of statistical significance for the difference in mean change from baseline between the nintedanib 150 mg bid and placebo groups.

^4^Treatment continued for ≥12 months (data not available).

Four studies [20,31,33,41] reported changes from baseline in SGRQ total score in the placebo group. Adjusting for different trial durations, the SGRQ total score in the placebo arms of these trials deteriorated (increased) by a median of +4.9 (range: 3.2 to 10.6) per 52 weeks.

### Floor and ceiling effects

No studies were found in which investigators reported data on floor and ceiling effects for the SGRQ in patients with IPF. However, in most studies, the minimum and maximum achievable SGRQ total scores (0 and 100, respectively) were outside an interval spanning twice the standard deviation around the reported means (Table 1). For the two studies in which investigators reported ranges for baseline SGRQ total scores, ranges did not include minimum or maximum possible values [24,38], thus confirming the absence of floor or ceiling effects in these studies.

## Conclusions

Measurement standards and psychometric criteria have been proposed to assist with choosing an appropriate instrument to evaluate HRQL in patients with IPF [6,43]. As with any patient-reported outcome measure used in the study of any condition, an instrument must have face validity, internal consistency, test-retest reliability, longitudinal validity, and minimal floor and ceiling effects in the target patient population.

The constellation of findings from studies identified in our search revealed that in patients with IPF, the internal consistency of the SGRQ activity and impact domains and the SGRQ total score was excellent, and the internal consistency of the symptoms domain was moderate, and in most studies, fell below the acceptable threshold of 0.7. The lower internal consistency of the symptoms domain is likely because it asks about a range of respiratory symptoms (cough, sputum, shortness of breath, wheezing and attacks of chest trouble), the majority of which apply to few patients with IPF whose major symptoms are shortness of breath and cough. In response data, off-target items create a weaker level of inter-relatedness among items in this domain, and thus lower internal consistency. This also contributes to the lower convergent validity of this domain, as the off-target items weaken the associations between its scores and clinical measures of IPF severity (e.g., patients may endorse wheezing or attacks of chest trouble, but these symptoms are unlikely related to a person’s FVC). These off-target (for IPF) items in the symptoms domain detract from the SGRQ’s face validity and would likely have been removed or modified in a tool specifically designed for use with patients with IPF. Overall, the symptoms domain may be well-suited for patients with COPD, but is not tailored to precisely assess symptoms in patients with IPF. The non-informative noise in the symptoms domain might also contribute to a less than optimal performance of the SGRQ total score. Overall, however, despite its weak face validity in IPF, the symptoms domain performs reasonably well in this population, and its potential to detract from the performance of the SGRQ total score is tempered because it contributes least to the SGRQ total score.

Convergent validity analyses seek to determine whether two measures, hypothesized to measure the same construct, do in fact correlate, and moderate, statistically significant correlations in the expected direction support convergent validity. Very strong or ‘perfect’ correlations, suggest redundancy in measurement, so moderate correlations between a patient-reported outcome measure and another clinical variable support convergent validity of the patient-reported outcome measure while confirming that it contributes unique information not captured by the other clinical variable [5]. The SGRQ has been used as a secondary endpoint in several clinical trials conducted in patients with IPF. Among the select few in which the intervention outperformed placebo, SGRQ results were as one would anticipate, i.e., SGRQ scores improved in the group that benefited from the intervention. Although not a formal assessment of responsiveness, consistency between the changes in SGRQ scores and the changes in other endpoints supports responsiveness.

In sum, the limitations of the SGRQ in IPF should be noted, as it was not originally developed for use in patients with IPF. In particular, this applies to possible over-interpretation of results of individual domains. However, the cross-sectional correlations between SGRQ domain and total scores and other measures of patient-reported health status, exercise capacity or lung function, along with the ability of the SGRQ to distinguish patients who experience a change in clinical status or remain stable over time, support the SGRQ as a useful patient-reported outcome measure in IPF.

Limitations to our research include the following: we could only identify one study in which MID estimates for the SGRQ scores in IPF were determined [44]. This study used a triangulation approach and concluded an MID that was higher than that reported for COPD [45], but more research with additional datasets is needed to evaluate these estimates. In the meantime, the use of responder rates of patients experiencing a minimum change from baseline in SGRQ scores – or perhaps more informative, cumulative distribution plots – may be a useful assessment, as research suggests that it may be less dependent on the exact cutoff, i.e. the precise value of the MID [46].

No articles were identified that evaluated the test-retest reliability of the SGRQ in patients with stable IPF. Likewise, we could not locate a study in which floor and ceiling effects of SGRQ scores were reported, although an analysis of the reported baseline mean SGRQ total scores and their standard deviations suggested that there was no evidence for either. Furthermore, we did not assess the content validity of the SGRQ in patients with IPF, nor did we include analyses of articles published in languages other than English. Content validity and cultural adaption are important factors to consider for any patient-reported outcome measure, but these topics were beyond the scope of this evaluation of the SGRQ’s psychometric properties. Therefore, it is evident that more research on the SGRQ is needed in this patient population.

The utility of a patient-reported outcome measure may be assessed only after a wealth of data becomes available. The assessment involves examining how the measure performs in the target population under several circumstances. The cache of available data has greatly advanced our understanding of HRQL in general, and the performance of the SGRQ in patients with IPF. For example, whilst the mean baseline SGRQ total score reported in IPF (around 45; interquartile range: 42–50) is similar to that reported in COPD trials [47,48], an analysis of the reported changes from baseline in the SGRQ total score in the placebo arms suggests that untreated patients with IPF deteriorate by +4.9 points over a period of 52 weeks. This contrasts with the experience in COPD, where patients on placebo show an improvement of 2–3 points per year [46], and reflects the progressive decline in health status seen in patients with IPF.

Finally, a major factor in this assessment revolves around how confidently response data from the measure can be used to make inferences about patients in the target population. For example, what can be said about a patient with IPF whose SGRQ score is 50? How does day-to-day functioning, or how a patient feels, change for an IPF patient whose SGRQ score increases by 10 over 6 months? Being able to answer these, and similar, questions confidently and accurately will further and more strongly support the validity of the SGRQ as an instrument capable of assessing domains of HRQL in this population. Until then, the balance of the data suggests that the SGRQ may be a suitable secondary endpoint for measuring HRQL in therapeutic trials of IPF.

## Additional file

Additional file 1:
**PubMed search strategy.**

## Abbreviations

6MWD: Distance covered in 6-minute walk test
BDI: Baseline Dyspnea Index
Bid: Twice daily
C: Comparator group
COPD: Chronic obstructive pulmonary disease
CQLQ: Cough Quality of Life Questionnaire
CT-alv: Degree of ground-glass opacity
CT-fib: Interstitial opacity
CT-tot: Total
D-12: Dyspnea-12
DL_CO_: Diffusing capacity for carbon monoxide
FEV_1_: Forced expiratory volume in 1 second
FVC: Forced vital capacity
HRCT: High-resolution computed tomography
HRQL: Health-related quality of life
IPF: Idiopathic pulmonary fibrosis
K-BILD: King’s Brief Interstitial Lung Disease questionnaire
MID: Minimal important difference
PaO_2_: Partial pressure of oxygen dissolved in arterial blood
PFT: Pulmonary function tests
Qd: Once daily
RCT: Randomized controlled trial
RVSD: Right ventricular systolic dysfunction
SF-36 PCS: Short Form-36 Physical Component Summary score
SGRQ: St George’s Respiratory Questionnaire
T: Treatment group
TDI: Transition dyspnea index
TLC: Total lung capacity
TL_CO_: Transfer factor of the lung for carbon monoxide
UCSD-SOBQ: University of California San Diego Shortness of Breath Questionnaire
VC: Vital capacity
