# Supplementary material for: The psychometric properties of the St George’s Respiratory Questionnaire (SGRQ) in patients with idiopathic pulmonary fibrosis: a literature review
Source: Health Qual Life Outcomes. 2014 Aug 20;12:124. doi: 10.1186/s12955-014-0124-1 (PMC4148554; doi:10.1186/s12955-014-0124-1)
Supplement: Additional file 1: — PubMed search strategy. [file 12955_2014_124_MOESM1_ESM.doc]

**Additional file 1: PubMed s**earch strategy

| Step | Search terms |
| --- | --- |
| #1 | “idiopathic pulmonary fibrosis”[MeSH] OR “IPF”[tiab] OR “pulmonary fibrosis”[MeSH] OR “pulmonary fibrosis” OR “lung diseases, interstitial”[MeSH] OR “interstitial lung disease*” OR “ILD”[tiab] OR “idiopathic interstitial pneumonia*" OR “IIP”[tiab] OR “idiopathic interstitial pneumonias” [MeSH] |
| #2 | "SGRQ"[Text Word] OR "St. George's Respiratory Questionnaire"[Text Word] OR "QOL"[Text Word] OR "quality of life"[Text Word] OR "patient reported outcome"[Text Word] OR "patient-reported outcome"[Text Word] |
| #3 | #1 AND #2 |
| #4 | (“idiopathic pulmonary fibrosis”[MeSH] OR “IPF”[tiab]) AND “Clinical Trial”[publication type] |
| #5 | #3 OR #4 |
| #6 | “animals”[MeSH] NOT “humans”[MeSH] |
| #7 | #5 NOT #6 |
